# Economic Impact of Biologics for Severe Asthma: A Case Series of 14 Patients From Northern Malaysia

**DOI:** 10.1002/rcr2.70447

**Published:** 2025-12-17

**Authors:** Arvindran Alaga, Izzatul Nadzirah Ismail

**Affiliations:** ^1^ Respiratory Department Hospital Sultanah Bahiyah Alor Setar Kedah Malaysia

**Keywords:** benralizumab, biologics, economic evaluation, omalizumab, severe asthma

## Abstract

There remains a paucity of evidence from Asia on the cost‐effectiveness of biologics for severe asthma. This pilot cost‐offset analysis aims to evaluate the economic impact of adding biologics (omalizumab or benralizumab) to severe asthma standard care as a proof of concept for its cost‐effectiveness, and to assess improvement in exacerbation rates and asthma control. Fourteen patients from the Severe Asthma Registry of Hospital Sultanah Bahiyah, Kedah who received biologics for at least 12 months from January 2018 to December 2024 were included. The economic impact of biologic treatment was evaluated by comparing the cost of biologic administration with the potential savings from reduced exacerbations requiring emergency department visits and hospital admission. Cost savings were observed with biologics, alongside reduced exacerbation and improved asthma control in real‐world settings. Future research could explore a full cost‐effectiveness analysis and a budget impact analysis to inform strategic pricing and reimbursement decisions.

## Graphical abstract

This pilot cost‐offset analysis serves as a proof of concept for evaluating the cost‐effectiveness of adding biologics to standard care in the management of severe asthma in Malaysia. In addition to cost savings, the use of biologics also reduces exacerbation and improves asthma control in real‐world settings.

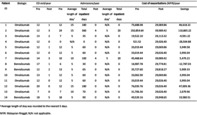

## 1 Introduction

Severe asthma is characterised by uncontrolled asthma despite adherence to optimised high‐dose inhaled corticosteroid and long‐acting β2 agonist therapy and addressing contributing factors, or worsening with reduction of high‐dose therapy [1]. Severe asthma represents around 5%–10% of asthma patients [2]. However, its management demands disproportionate healthcare resources and expenses [3], accompanied by substantial social costs arising from income loss and limited career opportunities [1]. These observations reinforce the critical need of implementing appropriate treatment approaches for severe asthma.

Real‐world studies support the cost‐effectiveness of biologics as an add‐on therapy for severe asthma [4, 5, 6, 7]. Nonetheless, there remains a paucity of evidence from Asia [8], particularly within the Malaysian healthcare system. Therefore, this pilot cost‐offset analysis aims to evaluate the economic impact of adding biologics (omalizumab or benralizumab) to standard care in the management of severe asthma as a proof of concept for its cost‐effectiveness, and to assess the exacerbation rates and asthma control following the addition of biologics.

## 2 Case Series

### 2.1 Methods

#### 2.1.1 Study Population and Study Design

This retrospective, cohort study analysed data from the Severe Asthma Registry of Hospital Sultanah Bahiyah, Kedah, located in Northern Malaysia. Patients aged 12 and above with severe asthma who were administered biologics (benralizumab or omalizumab) for at least 12 months from January 2018 to December 2024 were selected. Those below Age 12, and those with a diagnosis other than asthma (asthma mimickers), asthma‐chronic obstructive pulmonary disease overlap syndrome or evidence of malignancy were excluded from this study. As this study only involves anonymised data from the registry, ethics approval was not required.

The respiratory physician from the Respiratory Department of Hospital Sultanah Bahiyah, Alor Setar Team confirmed severe asthma diagnosis based on GINA's definition [1]. Additional initiation criteria for biologics include: (i) severe allergic, eosinophilic or mixed asthma; (ii) high immunoglobulin E (IgE) levels (≥ 150 IU/mL) or eosinophil counts (≥ 300 cells/μL) and (iii) two or more exacerbations during the previous year with the use of OCS despite receiving appropriate treatment, or corticosteroid dependence. Patients received benralizumab for severe eosinophilic asthma and omalizumab for severe persistent allergic asthma, in accordance with prescribing information [9, 10]. For those with mixed phenotype, biologics were selected based on predominant disease component.

#### 2.1.2 Statistical Analysis

The registry was compiled using the hospital medical records. Demographics data such as sex, age, comorbidities, asthma subtype and body mass index were recorded. Findings from high‐resolution computed tomography (HRCT) at first presentation were also documented. Clinical parameters, including IgE levels, eosinophil counts, spirometry data, fractional exhaled nitric oxide (FeNO) and asthma control test (ACT) scores, were measured at baseline and repeated during follow‐up visits after initiating biologics administration.

Statistical analysis was conducted using IBM SPSS Statistics for Windows, Version 31 (IBM Corp., Armonk, NY, USA). Categorical variables were expressed as counts and percentages; continuous variables were summarised using mean and standard deviation (SD) or median and interquartile range (IQR), where appropriate. Difference in clinical parameters between pre‐ and post‐biologics were tested using paired t‐test or Wilcoxon signed‐rank test, depending on data normality.

#### 2.1.3 Pilot Cost‐Offset Analysis

The economic impact of adding biologics to standard care in the management of severe asthma was evaluated by calculating the cost savings from reduced exacerbations requiring ED visits and hospital admission pre‐ and post‐biologics initiation. Calculation for annual cost of exacerbations considers: (i) costs of benralizumab (MYR 29,731.35/year) and omalizumab (MYR 29,026.40/year) based on 2023 hospital procurement, (ii) direct costs of ED visits (MYR 43.46/visit) and hospital admission (MYR 1777.86/5‐day admission) taken from Yong et al. micro‐costing [11] and (iii) indirect costs due to income lost (MYR 930.30/admission) from Lai et al. [12]. As all patients were employed, income loss during hospital stay was factored into the calculation.

The formula for annual cost of exacerbations and cost savings as follows:

$$\begin{array}{c} \text{Annual cost of exacerbations}\;\mathrm{pre}-\text{biologics initiation}\\ \;=\text{total cost of}\;\mathrm{ED}\;\text{visit}+\text{total cost of hospital admission}\\ \;+\text{income loss during hospital admission} \end{array}$$

$$\begin{array}{c} \text{Annual cost of exacerbations post}-\text{biologics initiation}\\ \;=\text{total cost of}\;\mathrm{ED}\;\text{visit}+\text{total cost of hospital admission}\\ \;+\text{income loss during hospital admission}\\ \;+\text{cost of biologics}\;\mathrm{per}\;\text{year} \end{array}$$

$$\begin{array}{c} \text{Cost savings from biologics initiation}\\ \;=\text{annual cost of exacerbations}\;\mathrm{pre}-\text{biologics initiation}\\ \;-\text{annual cost of exacerbations post}\\ \;-\text{biologics initiation} \end{array}$$

### 2.2 Results

Fourteen patients were identified from the Severe Asthma Registry. Patients reported a mean age of 47.2 (SD 18.2); most were females (78.6%). Half of the patients had mixed asthma subtype, followed by allergic (35.7%) and eosinophilic (14.3%) subtypes. Most patients (64.3%) were presented with normal HRCT findings.

Improvements in clinical parameters were outlined in Table 1. Following biologic intervention, median levels of IgE decreased from 586 IU/mL (IQR 785.75) to 143 IU/mL (IQR 276.5). However, this reduction was not statistically significant (*p* = 0.091, *r* = −0.12). Meanwhile, the mean blood eosinophil counts decreased from a baseline of 840 cells/μL (SD 347.9) to 206.4 cells/μL (SD 277.9) following biologics, resulting in a reduction of 633.6 cells/μL (*p* < 0.001, *d* = −2.02). More importantly, the mean ACT score improved from 10.4 pre‐biologics to 21.1 post‐biologics (*p* < 0.001), with a mean improvement of 102.9%. Biologics were well‐tolerated among all patients, with no treatment discontinuation due to adverse events.

**TABLE 1:** Clinical parameters pre‐ and post‐biologic initiation (*N* = 14).

Clinical parameters	Pre‐biologic initiation	Post‐biologic initiation	Paired difference	95% CI	Stat. value	*p*	Effect size
IgE (IU/mL), median (IQR)	586 (785.8)	143 (276.5)	−193	−657.0, 114.5	−1.69^a^	0.090^c^	−0.45^e^
Eosinophil count (cells/μL), mean (SD)	840.0 (347.9)	206.4 (277.9)	−633.6	−926.8, −340.4	−4.67 (13)^b^	< 0.001^d^	−2.02^f^
Spirometry, FEV1 (L), mean (SD)	1.6 (0.4)	1.7 (0.5)	0.1	−0.02, 0.2	1.75 (13)^b^	0.103^d^	0.17^f^
FeNO (ppb), median (IQR)	25.5 (38.8)	17.5 (17.3)	−7.0	−27.0, −5.0	−3.27^a^	0.001^c^	−0.87^e^
ACT score, mean (SD)	10.4 (2.4)	21.1 (3.8)	10.7	8.72, 12.71	11.62 (13)^b^	0.001^d^	3.26^f^

*Note:* Data for post‐biologic are taken from the latest follow‐up. Normality checks were done through inspection of histograms; eosinophil count, spirometry and ACT score were normally distributed, while IgE and FeNO were not.

Abbreviations: ACT, asthma control test; CI, confidence interval; FeNO, fractional exhaled nitric oxide; FEV1, forced expiratory volume in 1 s; IgE, immunoglobulin E; IQR, interquartile range; SD, standard deviation.

^a^ *Z* statistic.

^b^ *T*‐statistic (degree of freedom).

^c^ Wilcoxon Signed‐ranks test.

^d^ Paired *t*‐test.

^e^ Pearson's *r*.

^f^ Cohen's *d*.

A summary of exacerbations requiring ED visits and hospital admissions pre‐ and post‐biologic initiation, as well as the annual cost of exacerbations, is detailed in Table 2. Biologics therapy for severe asthma patients reduced ED visits from a median of 12 (IQR 1.8) to 1 (IQR 2) visit per year following biologics initiation (*p* = 0.001, r = −0.88). Similarly, exacerbation‐related hospital admissions also reduced from a mean of 11.2 (SD 5.6) to 0.6 (SD 1.5) episodes per year (*p* < 0.001, *d* = −2.2). Combined, this translates to a reduction on mean annual exacerbation (ED visits + hospital admissions) from 24.5 (SD 6.9) to 1.9 (SD 2.5) episodes per year (*p* < 0.001, *d* = −3.65). Reduced exacerbations requiring ED visits and hospital admission resulted in a mean cost‐saving of MYR 14,513.39 (equivalent to USD 3437.56; exchange rate: 1 USD = 4.22 MYR, 4 September 2025) per year per patient, reducing from a mean of MYR 45,297.35 (USD 10,733.97) to MYR 30,783.96 (USD 7794.78) per year.

**TABLE 2:** Exacerbations requiring ED visits and hospital admissions pre‐ and post‐biologic initiation, as well as the annual cost of exacerbations (*N* = 14).

Patient ID	Biologic	ED visit/year		Admissions/year						Cost of exacerbations (MYR)/year		
		Pre	Post	Pre	Average length of stay^a^	Total inpatient days	Post	Average length of stay^a^	Total inpatient days	Pre	Post	Savings
1	Omalizumab	12	1	12	15	180	0	N/A	0	75,688.08	29,069.86	46,618.22
2	Omalizumab	12	3	24	15	360	4	5	20	150,854.64	39,989.42	110,865.22
3	Omalizumab	13	2	7	5	35	0	N/A	0	19,522.10	29,113.32	−9591.22
4	Omalizumab	12	0	0	N/A	0	0	N/A	0	521.52	29,026.40	−28,504.88
5	Omalizumab	12	1	12	5	60	0	N/A	0	33,019.44	29,069.86	3949.58
6	Omalizumab	12	0	12	5	60	0	N/A	0	33,019.44	29,026.40	3993.04
7	Omalizumab	14	3	10	10	100	4	5	20	45,468.64	39,989.42	5479.22
8	Benralizumab	17	1	6	5	30	0	N/A	0	16,987.78	29,774.81	−12,787.03
9	Benralizumab	12	2	13	5	65	0	N/A	0	35,727.60	29,818.27	5909.33
10	Omalizumab	13	1	12	5	60	0	N/A	0	33,062.90	29,069.86	3993.04
11	Omalizumab	12	0	12	5	60	0	N/A	0	33,019.44	29,026.40	3993.04
12	Omalizumab	20	0	12	15	180	0	N/A	0	76,035.76	29,026.40	47,009.36
13	Omalizumab	7	0	7	10	70	0	N/A	0	31,706.36	29,026.40	2679.96
14	Benralizumab	18	5	18	5	90	0	N/A	0	49,529.16	29,948.65	19,580.51

Abbreviations: MYR, Malaysian Ringgit; N/A, not applicable.

^a^ Average length of stay was rounded to the nearest 5 days.

## 3 Discussion

This pilot cost‐offset analysis serves as a proof of concept for evaluating the cost‐effectiveness of adding biologics to standard care in the management of severe asthma in Malaysia. The addition of biologics resulted in a significant reduction in annual exacerbations, with the mean decreasing from 24.5 to 1.9 episodes per year, and better asthma control, as evidenced by an increase in mean ACT score from 10.4 to 21.1. These improvements led to a substantial mean cost‐saving of MYR 14,513.39 (USD 3437.56) per year per patient after at least a year of biologics initiation.

Biologics therapy has resulted in notable clinical improvements in patients with severe asthma that remain uncontrolled despite optimal standard treatment. Landmark studies on benralizumab (SIROCCO and CALIMA) [13, 14] and omalizumab (INNOVATE, EXALT and EXTRA) [15, 16, 17] show that add‐on biologic therapy reduces exacerbation, improves lung function and symptoms, and sustains benefits long‐term without new safety concerns [18, 19, 20, 21]. Despite only having two biologics available for this initial cohort, our findings align with previous studies, indicating significant reductions in annual exacerbations, along with significant improvements in both FEV1 and ACT score; good tolerability was observed, with no treatment discontinuation due to adverse events. Considering the increasing availability of biologics in Malaysia, this underscores the need for timely discussions on optimising asthma control.

While biologic therapies have emerged as a promising intervention for severe asthma, their full benefit can only be realised if they are accessible. The addition of biologics to standard asthma therapy mainly incurs costs to the drug itself and its administration, but these may be offset by potential savings from reduced hospitalisation, ED visits and primary care utilisation [22, 23]. The European Academy of Allergy and Clinical Immunology (EAACI) Guidelines on biologic use in severe asthma reported an incremental cost‐effectiveness ratio (ICER) of approximately €30,000 per quality‐adjusted life‐year (QALY), positioning biologics above the willingness‐to‐pay (WTP) threshold in most European countries [22]. The 2024 Malaysian Health Technology Assessment report on biologics for severe asthma reported similar findings, where ICERs from all included studies exceeded the Malaysian WTP threshold of one gross domestic product (GDP) per capita per QALY gained based on 2022 GDP of MYR 54,863 per QALY, highlighting their limited cost‐effectiveness within the local healthcare context [23].

Enhancing the cost‐effectiveness of biologics requires careful balancing between policymaker priorities and pharmaceutical pricing strategies. When prescribed for appropriately selected patients, the high cost per vial of biologics was identified as a key contributor to the unfavourable ICER, which would require substantial price reductions to achieve cost‐effectiveness [23]. Although biologic therapies are clinically effective, an ICER per QALY exceeding the WTP threshold emphasises the need for effective price negotiations to improve their cost‐effectiveness [23]. This is imperative, as patients who meet the criteria for biologic therapy but are unable to access it continue to bear a high disease burden, where 49.6% experience at least two exacerbations and 71.2% have uncontrolled asthma in the past 12 months [24]. Strategic policies that align cost with established clinical value are key to enabling broader and sustainable access to these life‐changing therapies.

This project provides a proof of concept on its cost‐effectiveness within the Malaysian public healthcare setting. Considering that this pilot cost‐offset analysis involved a small sample of 14 patients from a single tertiary hospital in Northern Malaysia, expanding the study across multiple centres and states would enhance the generalisability and robustness of the findings. Building on the prior micro‐costing study [11], a full cost‐effectiveness analysis would offer greater clarity on the long‐term value of biologics compared to standard care. In parallel, a budget impact analysis could estimate the financial implications of scaling up biologic use within the public healthcare system. Together, these analyses provide a strategic framework for price negotiation, balancing clinical value with fiscal feasibility to enable the adoption of biologics in Malaysia.

In conclusion, this pilot cost‐offset analysis serves as a proof of concept for evaluating the cost‐effectiveness of adding biologics to standard care in the management of severe asthma in Malaysia, indicating that the addition of biologics results in cost savings while showing reduced exacerbation and improved asthma control in real‐world settings. Future research could explore a full cost‐effectiveness analysis alongside a budget impact analysis to inform strategic pricing and reimbursement decisions regarding the adoption of biologics in Malaysia.

## Author Contributions

A.A. conceptualised the study, reviewed and edited the draft, secured funding for medical writing services and supervised the project. I.N.I. extracted data from the Severe Asthma Registry, conducted the analysis and prepared and reviewed the draft. All authors approved the final version of the manuscript and agreed to be accountable for all aspects of the work.

## Funding

This academic project undertaken by Pertubuhan Perubatan Respiratori Kedah was kindly supported by an unrestricted educational grant from AstraZeneca Malaysia for medical writing assistance and publication‐related expenses.

## Ethics Statement

As this study only involves anonymised data from the Severe Asthma Registry, which does not contain identifiable patient information, ethics approval was not required.

## Consent

As this study only involves anonymised data from the Severe Asthma Registry, which does not contain identifiable patient information, consent for publication was not required.

## Conflicts of Interest

A.A. has received honorarium as speaker from Astra Zeneca and Menarini; A.A. also served in the advisory board for *Respirology Case Report*, he was excluded from all editorial decision‐making related to the acceptance of this article for publication. I.N.I. has received the Assembly Award in the 2024 APSR Conference for this topic.

## Data Availability

The data that support the findings of this study are available on request from the corresponding author. The data are not publicly available due to privacy or ethical restrictions.
